# Highly Efficient Infrared Photodetection in a Gate‐Controllable Van der Waals Heterojunction with Staggered Bandgap Alignment

**DOI:** 10.1002/advs.201700423

**Published:** 2018-01-18

**Authors:** Seo‐Hyeon Jo, Hae Won Lee, Jaewoo Shim, Keun Heo, Minwoo Kim, Young Jae Song, Jin‐Hong Park

**Affiliations:** ^1^ School of Electronic and Electrical Engineering Sungkyunkwan University Suwon 16419 South Korea; ^2^ SKKU Advanced Institute of Nano Technology (SAINT) Sungkyunkwan University Suwon 16419 South Korea

**Keywords:** heterojunctions, infrared detectors, interlayer optical transitions, photodetectors, van der Waals materials

## Abstract

In recent years, various van der Waals (vdW) materials have been used in implementing high‐performance photodetectors with high photoresponsivity over a wide detection range. However, in most studies reported so far, photodetection in the infrared (IR) region has not been achieved successfully. Although several vdW materials with narrow bandgaps have been proposed for IR detection, the devices based on these materials exhibit notably low photoresponsivity under IR light illumination. Here, highly efficient near‐infrared (NIR) photodetection based on the interlayer optical transition phenomenon in a vdW heterojunction structure consisting of ReS_2_ and ReSe_2_ is demonstrated. In addition, by applying the gate‐control function to the two‐terminal vdW heterojunction photodetector, the photoresponsivity is enhanced to 3.64 × 10^5^ A W^−1^ at λ = 980 nm and 1.58 × 10^5^ A W^−1^ at λ = 1310 nm. Compared to the values reported for previous vdW photodetectors, these results are the highest levels of photoresponsivity in the NIR range. The study offers a novel device platform for achieving high‐performance IR photodetectors.

## 1 Introduction

Since the graphene photodetector was first implemented in 2009,^1^ various van der Waals (vdW) materials, such as graphene,^1^, ^2^, ^3^, ^4^ transition metal dichalcogenides (TMDs),^5^, ^6^, ^7^, ^8^, ^9^, ^10^, ^11^, ^12^ and black phosphorus (BP),^13^, ^14^, ^15^ have been utilized to achieve high‐performance photodetectors with high photoresponsivity and a wide detection range. In the early graphene‐based photodetectors, photodetection in a wide range from ultraviolet to terahertz wavelengths was possible, owing to the zero‐bandgap nature of graphene.^16^ However, this zero‐gap nature worked negatively in terms of photocarrier lifetime and electron–hole recombination, thereby decreasing the collection probability of photocarriers.^17^ As a result, graphene photodetectors with broad detection ranges have presented relatively low photoresponsivity values between 10^−6^ and 10 A W^−1^.^1^, ^2^, ^3^, ^4^ After that, by replacing graphene with TMD materials having moderate energy bandgaps between 1 and 2 eV, the photoresponsivity of the vdW photodetector was further enhanced.^18^, ^19^ Although the photoresponsivity values of photodetectors based on group VI‐TMDs (e.g., MoS_2_, MoSe_2_, WS_2_, and WSe_2_) and group VII‐TMDs (e.g., ReS_2_ and ReSe_2_) were reported to be between 10^4^ and 10^7^ A W^−1^ under exposure to light with λ = 520 nm,^5^, ^6^, ^7^, ^8^, ^10^, ^20^, ^21^, ^22^ it was not easy to detect infrared (IR) light with a wavelength greater than 900 nm in most of the TMD photodetectors owing to their energy bandgap properties.^18^, ^19^ In light of this limitation, the ternary metal chalcogenide synthesis technique, which can be used to design various energy band structures through stoichiometric alteration, has recently been proposed for IR photodetection.^23^, ^24^ The narrow energy bandgaps of such designed ternary metal chalcogenides enabled vdW photodetectors to operate in a considerably wider spectrum range.^23^, ^24^ However, compared to the conventional TMD‐based devices, these vdW photodetectors exhibited relatively low photoresponsivity between 3 × 10^−1^ and 6 × 10^3^ A W^−1^.^23^, ^24^, ^25^, ^26^, ^27^ This is because of the poor crystallinity resulting from the complicated synthesis process that needs to precisely control the relative quantities of the three different constituent atoms.^27^, ^28^ In the case of recently reported BP with an intermediate bandgap (0.35 eV) between graphene and TMDs, it was possible to detect lights in a broader wavelength range. The BP devices also exhibited appreciably higher photoresponsivity values between 5 × 10^−3^ and 9 × 10^4^ A W^−1^.^13^, ^14^, ^15^ However, it was difficult to use BP for the fabrication of vdW photodetectors because BP is highly hygroscopic and tends to condense moisture on its surface, and consequently being well decomposed in air.^29^

Overall, in most studies on vdW photodetectors reported so far, there was difficulty in achieving high photoresponsivity and IR range detection simultaneously. Although several vdW materials with narrow bandgaps have been recently utilized for IR detection, the photodetectors based on these materials showed serious drawbacks, such as low photoresponsivity, slow response speed, and environmental instability. Here, we demonstrate a highly efficient NIR photodetector based on the interlayer optical transition phenomenon at the heterojunction interface of vdW materials. In particular, by adding a gate‐control function to a two‐terminal vdW NIR photodetector, we control the heterojunction interface and maximize the photoresponsivity in the NIR range. First, we investigate the role of metal contacts (Ti and Pt) on the performance of single‐vdW‐material‐based photodetectors (ReS_2_ and ReSe_2_). Then, we implement a ReS_2_/ReSe_2_ heterojunction with a staggered bandgap alignment, where the interlayer optical transition phenomenon occurs, and confirm this bandgap alignment using Kelvin probe force microscopy (KPFM). Finally, we fabricate a gate‐controllable ReS_2_/ReSe_2_ heterojunction photodetector and analyze its capability of photodetection up to the NIR spectral range.

## 2 Results and Discussion

**Figure 1:**
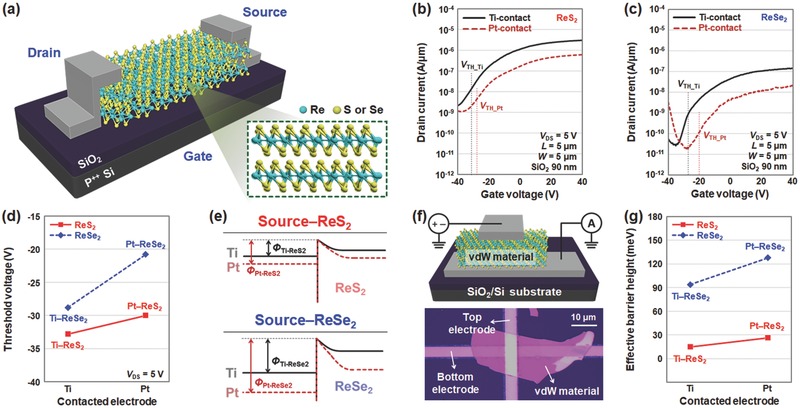
Electronic device characterization of ReS_2_ and ReSe_2_ transistors. a) Schematic of the back‐gated transistor fabricated on a vdW material (ReS_2_ and ReSe_2_). *I*_D_–*V*_G_ characteristics of b) ReS_2_ and c) ReSe_2_ transistors with Ti‐contact (black solid line) or Pt‐contact (red dotted line). d) Extracted threshold voltage of ReS_2_‐ (red solid line) and ReSe_2_‐ (blue dotted line) based transistors with Ti‐ or Pt‐contact. e) Energy band diagrams of source‐ReS_2_ (top) and source‐ReSe_2_ (bottom) junctions with Ti‐contact (black solid line) or Pt‐contact (red dotted line). f) Schematic and optical image of the metal–vdW material–metal (MVM) junction device. g) Calculated effective barrier height values in MVM junction devices with Ti‐ or Pt‐contacts.

To understand the roles of metal contacts on the performance of ReS_2_‐ and ReSe_2_‐based electronic devices, we fabricated back‐gated field‐effect transistors (FETs) with two different metal contacts (Ti and Pt) and then performed electrical measurements. **Figure** **1**a presents the schematic of a FET device (both channel length and width are 5 µm) fabricated using a vdW material. According to the *I*_D_–*V*_G_ curves (at *V*_DS_ = 5 V) of the ReS_2_‐ and ReSe_2_‐based devices (Figure 1b,c), the on‐current (*I*_on_) and threshold voltage (*V*_TH_) were higher and lower, respectively, in the Ti‐contacted devices, as compared to the Pt‐contacted devices. As shown in Figure 1d, Ti‐contacted ReS_2_ (*V*_TH_Ti–ReS2_: −32.8 V) and ReSe_2_ (*V*_TH_Ti–ReSe2_: −28.8 V) devices both have smaller threshold voltages than Pt‐contacted ReS_2_ (*V*_TH_Pt–ReS2_: −30.0 V) and ReSe_2_ (*V*_TH_Pt–ReSe2_: −20.8 V) devices, respectively. Owing to the smaller work function of Ti (*W*_Ti_ = 4.33 eV < *W*_Pt_ = 6.35 eV), relatively low electron barriers (Φ_Ti_) are formed at the junctions between Ti and vdW materials, compared to Pt–vdW junctions. These lower barriers increase the probability of electron injection from the source metal to vdW materials, thereby reducing *V*_TH_ and increasing *I*_on_ in both the Ti‐contacted devices (Figure 1e). Additionally, for the same contact metal, when compared to the ReSe_2_ devices (Figure 1c), the ReS_2_ devices (Figure 1b) required a smaller gate voltage (*V*_GS_) bias to turn on the device (*V*_TH_ReS2_ < *V*_TH_ReSe2_) and consequently exhibited a higher on‐current (*I*_on_ReS2_ > *I*_on_ReSe2_), although ReSe_2_ has a smaller energy bandgap than ReS_2_.

To quantitatively analyze the difference in the barrier heights between metals (Ti and Pt) and vdW materials (ReS_2_ and ReSe_2_), we performed temperature‐dependent electrical measurements on the metal–vdW material–metal (MVM) junction devices. Figure 1f shows the schematic and optical image of the device with a junction area of ≈25 µm^2^. In this study, we investigated the *I*–*V* characteristics at various temperatures and then extracted the effective barrier height values using a conventional thermionic emission model (Figure S1, Supporting Information). The obtained effective barrier heights are plotted in Figure 1g. As expected, the Ti‐contacted ReS_2_ (Φ_eff_Ti–ReS2_: 14.9 meV) and ReSe_2_ (Φ_eff_Ti–ReSe2_: 93.7 meV) devices presented smaller effective barrier heights than the Pt‐contacted devices (Φ_eff_Pt–ReS2_: 26.5 meV and Φ_eff_Pt–ReSe2_: 127 meV). This is consistent with the studies shown in Figure 1b,c, where higher on‐current and smaller threshold voltage values were confirmed in Ti‐contacted devices. In this experiment, we also found that the metal–ReS_2_ junctions had lower effective barrier height (Φ_eff_ReS2_) values, compared to those (Φ_eff_ReSe2_) of the metal–ReSe_2_ junctions. The Fermi level pinning phenomenon probably causes the relatively low electron barrier heights in the metal–ReS_2_ junctions, thereby reducing the gate voltage bias required to turn on the ReS_2_ device and providing higher on‐current, compared to ReSe_2_ devices (refer to Figure 1b,c).

**Figure 2:**
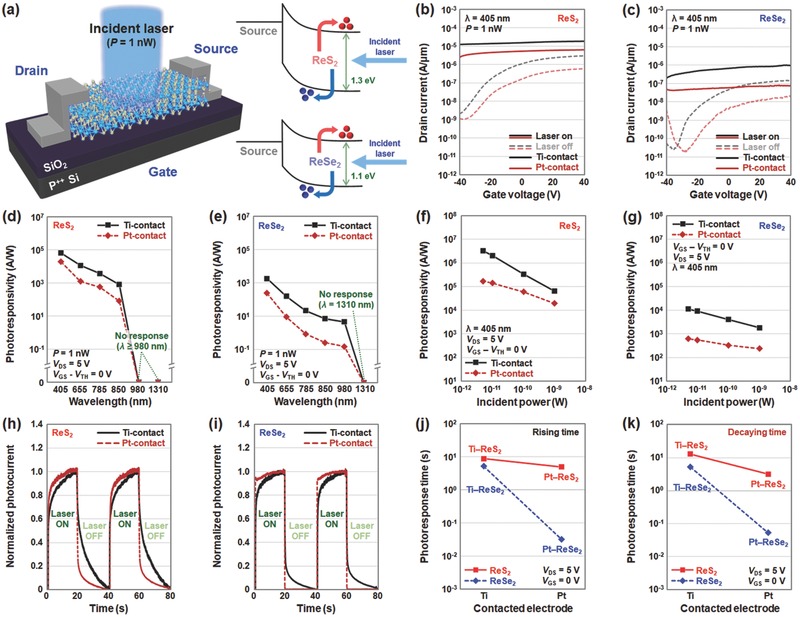
Optoelectronic device characterization of ReS_2_ and ReSe_2_ photodetectors. a) Schematic of photodetector fabricated on vdW material under laser illumination and energy band diagrams of source–vdW junctions. *I*_D_–*V*_G_ characteristics of b) ReS_2_ and c) ReSe_2_ photodetectors under dark (dotted line) and illuminated (solid line) conditions with Ti‐contact (black line) or Pt‐contact (red line). Extracted photoresponsivity as a function of the wavelength in the d) ReS_2_ and e) ReSe_2_ photodetectors with Ti‐contact (black solid line) or Pt‐contact (red dotted line). Photoresponsivity as a function of the incident laser power obtained at the f) ReS_2_ and g) ReSe_2_ photodetectors with Ti‐contact (black solid line) or Pt‐contact (red dotted line). Normalized temporal photoresponse curves of the h) ReS_2_ and i) ReSe_2_ photodetectors with Ti‐contact (black solid line) or Pt‐contact (red dotted line). Extracted j) rising and k) decaying times of the ReS_2_ (red solid line) and ReSe_2_ (blue dotted line) photodetectors with Ti‐ or Pt‐contacts.

Subsequently, we performed electrical measurements under dark and laser illuminated conditions for the ReS_2_ and ReSe_2_ devices with two different metal contacts (Ti and Pt) to investigate the effects of the contact metals in terms of optoelectronic device performance. **Figure** **2**a shows the schematic of the vdW photodetector and the energy band diagrams of junctions between the source metal and vdW materials, where ReS_2_ (1.3 eV^30^) has a larger optical bandgap than ReSe_2_ (1.1 eV^30^). As seen in Figure 2b,c, the Ti‐contacted devices showed higher photocurrent values (*I*_ph_Ti–ReS2_: 1.29 × 10^−5^ A µm^−1^ and *I*_ph_Ti–ReSe2_: 3.62 × 10^−7^ A µm^−1^) than Pt‐contacted ReS_2_ (*I*_ph_Pt–ReS2_: 3.87 × 10^−6^ A µm^−1^) and ReSe_2_ (*I*_ph_Pt–ReSe2_: 4.77 × 10^−8^ A µm^−1^) devices, respectively. This is because the relatively low contact resistances of Ti‐contacted devices induce a larger electric field inside the channel region, thereby increasing the collection probability of photocarriers. A detailed explanation with corresponding energy band diagrams is provided in Figure S2 (Supporting Information). In this study, the photocurrents were extracted under the following bias conditions: *V*_GS_ = *V*_TH_ and *V*_DS_ = 5 V. The difference between the photocurrent values of the ReS_2_ and ReSe_2_ photodetectors was greater than one order of magnitude (*I*_ph_ReS2_ > *I*_ph_ReSe2_). This can be explained by the previous analysis result that the ReS_2_ device shows a lower effective barrier height (Φ_eff_ReS2_ < Φ_eff_ReSe2_). Next, we compared the extracted photoresponsivity values according to the various wavelength (Figure 2d,e) and power (Figure 2f,g) conditions of the incident lasers. Under laser irradiation by wavelengths less than 850 nm, the ReS_2_ photodetector clearly showed photoresponses, but no responses were observed at the 980 and 1310 nm wavelengths (Figure 2d). On the other hand, in the ReSe_2_ photodetector, we confirmed a relatively broader photodetection range (up to 980 nm) compared to that of the ReS_2_ device (Figure 2e), owing to the smaller bandgap of ReSe_2_ (Figure 2a). Additionally, for both ReS_2_ and ReSe_2_, the Ti‐contacted devices exhibited higher photoresponsivity values over the entire wavelength range than those of Pt‐contacted devices. The photoresponsivity values were also analyzed as a function of incident laser power in Figure 2f,g. In both ReS_2_ and ReSe_2_ photodetectors, an exponential increase in photoresponsivity was observed with a decrease in the incident laser power. This is because the scattering phenomenon among the photocarriers was suppressed under low laser power conditions.^12^ The maximum photoresponsivity values were 3.23 × 10^6^ and 1.13 × 10^4^ A W^−1^ that were obtained in the Ti‐contacted ReS_2_ and ReSe_2_ photodetectors, respectively, under 5 pW laser illumination.

Furthermore, we investigated the temporal photoresponse characteristics of the vdW photodetectors that were measured by repeatedly turning on and off the laser with a switching cycle of 20 s. Figure 2h,i presents the temporal photoresponse curves for the ReS_2_ and ReSe_2_ photodetectors, respectively. Compared to Pt‐contacted devices, a relatively slow photoresponse was observed in Ti‐contacted photodetectors at both the rising and decaying edges. The extracted rising and decaying times are plotted in Figure 2j,k, and the extraction method is explained in detail in Figure S3 (Supporting Information). According to this photoresponse data, the rising/decaying times of the Ti‐contacted photodetectors (8.74/13.1 s for ReS_2_ and 5.20/5.30 s for ReSe_2_) were considerably longer than those of the Pt‐contacted devices (4.94/3.20 s for ReS_2_ and 0.032/0.053 s for ReSe_2_). This is probably attributable to the difference in depletion widths at the junctions between metals and vdW materials. When Ti is used as a contact metal, the depletion width is expected to be shortened, subsequently making it more difficult to quickly collect photocarriers. The relationship between the contact metal and rising/decaying times has been explained in more detail by adding energy band diagrams (Figure S2, Supporting Information). In addition, in terms of vdW materials, the photoresponse of ReSe_2_ photodetectors was considerably more rapid than that of ReS_2_ photodetectors, because of the different types of chalcogen vacancies in the respective vdW materials. Here, the selenium vacancies serving as traps in ReSe_2_ seem to possess considerably shorter recombination and generation times for carriers than the sulfur vacancies in ReS_2_. This result agrees well with previous studies on vdW photodetectors, in which considerably shorter photoresponses were confirmed in selenide‐based devices compared to sulfide‐based devices.^7^, ^31^ Additionally, we investigated the stability of the photodetectors with respect to the repetitive photoswitching cycle (Figure S4, Supporting Information). During ten photoswitching cycles, the photoresponse characteristics were continuously monitored, and no photocurrent degradation was observed.

Although the Ti‐contacted ReS_2_ and ReSe_2_ photodetectors exhibited high photoresponsivity values (3.23 × 10^6^ A W^−1^ for ReS_2_ and 1.13 × 10^4^ A W^−1^ for ReSe_2_), these devices showed slightly slow photoresponse properties (rising/decaying times: 8.74/13.1 s for ReS_2_ and 5.2/5.3 s for ReSe_2_). In the case of Pt‐contacted photodetectors, relatively rapid photoresponse characteristics were observed (rising/decaying times: 4.94/3.20 s for ReS_2_ and 0.032/0.053 s for ReSe_2_), but the photoresponsivity values were considerably lower, owing to the high contact resistance between the metal and vdW materials (refer to Figures 1 and 2). In addition, the ReS_2_ and ReSe_2_ devices presented the limited photodetection characteristic in terms of the wavelength of light (up to 850 nm for ReS_2_ and 980 nm for ReSe_2_), according to their energy bandgap properties. Therefore, in this experiment, to broaden the detection range of these ReS_2_‐ and ReSe_2_‐based photodetectors, we utilized a heterojunction structure consisting of ReS_2_ and ReSe_2_. Recently, vdW heterojunctions have been proposed for the implementation of high‐performance optoelectronic devices, because these heterojunctions exhibit novel and excellent functionalities (e.g., long‐lived interlayer excitons,^32^ ultrafast charge transfer,^33^ and interlayer optical transitions^34^) in comparison with individual vdW materials.

**Figure 3:**
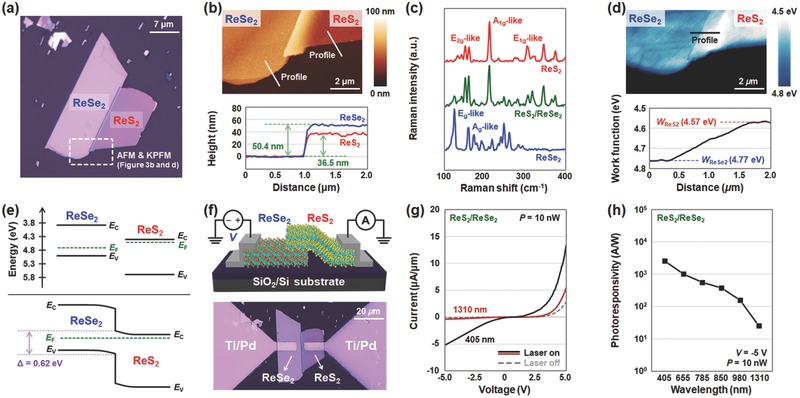
Characterization of ReS_2_/ReSe_2_ heterojunction. a) Optical image of the ReS_2_/ReSe_2_ heterojunction structure. b) AFM mapping images and height profiles of ReS_2_ (red line) and ReSe_2_ (blue line). c) Raman spectra of ReS_2_ (red line), ReSe_2_ (blue line), and overlapped region (green line) in the ReS_2_/ReSe_2_ heterojunction structure. d) KPFM mapping image and work function profile obtained on the surface of ReS_2_/ReSe_2_ heterojunction structure. e) Energy band diagrams of the ReS_2_/ReSe_2_ heterojunction at equilibrium before (top) and after contact (bottom). f) Schematic and optical image of ReS_2_/ReSe_2_ heterojunction device. g) *I*–*V* characteristics of the ReS_2_/ReSe_2_ heterojunction device under dark (gray dotted line) and laser illuminated (black and red solid lines) conditions (λ = 405 and 1310 nm). h) Photoresponsivity as a function of the wavelength obtained at the ReS_2_/ReSe_2_ heterojunction device.

**Figure** **3**a presents an optical image of the ReS_2_/ReSe_2_ heterojunction structure formed on the SiO_2_/Si substrate. The ReSe_2_ layer was first prepared on an SiO_2_/Si substrate using a tape‐based exfoliation method, and then the ReS_2_ layer was transferred onto the ReSe_2_ layer by a mechanical transfer process.^35^ The thicknesses of the ReS_2_ and ReSe_2_ layers were confirmed to be 36.5 and 50.4 nm by atomic force microscope (AFM), respectively (Figure 3b). Figure 3c shows the Raman spectra obtained at three different positions: the ReS_2_ region (red line), the ReSe_2_ region (blue line), and the overlapped region (green line) in the ReS_2_/ReSe_2_ heterojunction structure. In the ReS_2_ region, the Raman peaks observed at 152 and 212 cm^−1^ are related to the E_2g_‐like and A_1g_‐like phonon modes, respectively.^36^ The two peaks with high signal intensities in the ReSe_2_ Raman spectrum (at 125 and 158 cm^−1^) indicate E_g_‐like and A_g_‐like Raman peaks.^37^ The Raman spectrum of the overlapped ReS_2_/ReSe_2_ region contained all peaks related to the vibration modes of both ReS_2_ and ReSe_2_. Subsequently, we performed KPFM analysis to verify the energy band alignment of the ReS_2_/ReSe_2_ heterojunction. Prior to measurement, the KPFM tip was calibrated on a highly oriented pyrolytic graphite (HOPG) surface (this is explained in detail in the Experimental Section). Figure 3d presents the KPFM mapping image of the ReS_2_/ReSe_2_ heterojunction and the extracted work function profile, where the work function values of the ReS_2_ and ReSe_2_ layers were confirmed to be 4.57 and 4.77 eV, respectively. We show the energy band alignment of the ReS_2_/ReSe_2_ heterojunction (Figure 3e) on the basis of the KPFM data and the previously reported band edge properties (conduction band minimum (CBM) and valence band maximum (VBM)) of ReS_2_ and ReSe_2_.^30^ This ReS_2_/ReSe_2_ heterojunction forms a staggered bandgap alignment (type‐II heterojunction) with an interlayer gap of 0.62 eV between the VBM of ReSe_2_ and the CBM of ReS_2_. After the formation of contact electrodes on the ReS_2_/ReSe_2_ heterojunction, we performed electrical measurements under both dark and laser illuminated conditions. Figure 3f shows the schematic and optical image of the ReS_2_/ReSe_2_ heterojunction device. Under both visible (λ = 405 nm) and NIR (λ = 1310 nm) light illumination, we observed photocurrents of 5.05 × 10^−6^ and 4.98 × 10^−8^ A µm^−1^, respectively, at *V* = −5 V (Figure 3g). For reference, no photoresponse to light with a wavelength of 1310 nm was observed in previous photodetector devices formed on individual ReS_2_ and ReSe_2_ (Figure 2d,e). The extension of the photodetection range to the NIR region is a result of the interlayer optical transition between the VBM of ReSe_2_ and the CBM of ReS_2_, which requires less energy than the band‐to‐band transition of individual materials. Considering the interlayer gap of 0.62 eV, the photodetection in the ReS_2_/ReSe_2_ heterojunction device is expected to be feasible up to 2000 nm. We next extracted the photoresponsivity values according to the wavelengths of the incident light, as shown in Figure 3h. The ReS_2_/ReSe_2_ heterojunction device exhibited excellent photoresponse characteristics over the entire wavelength range, particularly by extending the cut‐off wavelength to greater than 1310 nm, which could not be covered by previous photodetectors fabricated on individual ReS_2_ and ReSe_2_ (Figure 2d,e).

**Figure 4:**
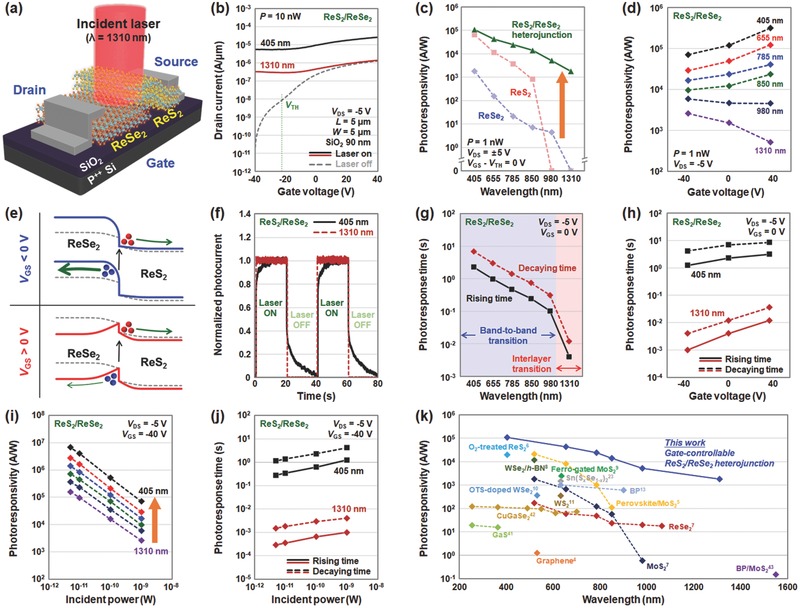
Optoelectronic device characterization of gate‐controllable ReS_2_/ReSe_2_ heterojunction photodetector. a) Schematic of the gate‐controllable ReS_2_/ReSe_2_ heterojunction photodetector. b) *I*_D_–*V*_G_ characteristics of the ReS_2_/ReSe_2_ photodetector under dark (gray dotted line) and illuminated (black and red solid lines) conditions (λ = 405 and 1310 nm). c) Photoresponsivity as a function of the wavelength obtained in the photodetectors fabricated on ReS_2_ (red dotted line), ReSe_2_ (blue dotted line), and the ReS_2_/ReSe_2_ heterojunction (green solid line). d) Extracted photoresponsivity as a function of gate voltage (*V*_GS_ = −40, 0, 40 V) obtained in the ReS_2_/ReSe_2_ heterojunction device under various wavelength conditions. e) Energy band diagrams of the ReS_2_/ReSe_2_ heterojunction under negative gate voltage (top) and positive gate voltage (bottom) biases. f) Normalized temporal photoresponse curves and g) extracted photoresponse times (rising/decaying times) for the ReS_2_/ReSe_2_ heterojunction photodetector under various wavelength conditions. h) Extracted rising (solid line) and decaying (dotted line) times according to the gate voltage (*V*_GS_ = −40, 0, 40 V) of the ReS_2_/ReSe_2_ heterojunction device under visible (black line) and NIR (red line) light illuminated conditions (λ = 405 and 1310 nm). i) Photoresponsivity values and j) photoresponse times as a function of the incident laser power obtained in the ReS_2_/ReSe_2_ heterojunction device under various wavelength conditions. k) Photoresponsivity as a function of wavelength obtained for the ReS_2_/ReSe_2_ heterojunction photodetector and values reported in studies for vdW photodetectors.

Furthermore, by additionally providing a gate‐control functionality, we fabricated a gate‐controllable ReS_2_/ReSe_2_ heterojunction photodetector. We then analyzed how the photoresponse characteristics of this device depend on the gate voltage bias. **Figure** **4**a shows the schematic of a ReS_2_/ReSe_2_ heterojunction photodetector, which can be controlled by a back‐gate bias. As shown in Figure 4b, we observed n‐channel transistor behavior in the ReS_2_/ReSe_2_ heterojunction device, where the photoresponse became more obvious as a higher negative gate bias was applied, owing to an exponential decrease in dark current. In the off‐state (*V*_GS_ = −40 V), when the device was irradiated by visible (λ = 405 nm) and NIR (λ = 1310 nm) light, the photocurrents were 5.55 × 10^−6^ and 3.27 × 10^−7^ A µm^−1^, respectively. From the *I*_D_–*V*_G_ curves in Figure 4b, we then extracted the photoresponsivity values for the ReS_2_/ReSe_2_ heterojunction photodetectors according to the various wavelengths of the incident lasers (Figure 4c). The obtained values (green solid line) were compared with the photoresponsivity of the devices that were previously fabricated on ReS_2_ (red dotted line) and ReSe_2_ (blue dotted line). We note that these photoresponsivity values were extracted under the same gate bias conditions (*V*_GS_ − *V*_TH_ = 0 V). In the case of the ReS_2_/ReSe_2_ heterojunction photodetector, relatively higher photoresponsivity values were confirmed over the entire wavelength range from the visible to NIR region, compared to both the ReS_2_ and ReSe_2_ photodetectors. In particular, this heterojunction photodetector exhibited considerably higher photoresponsivity under NIR (λ = 980 and 1310 nm) light illumination, while significant degradations in photoresponsivity were observed at wavelengths greater than 850 nm (no response at 980 and 1310 nm) in the ReS_2_ device and at wavelengths greater than 980 nm (no response at 1310 nm) in the ReSe_2_ device. The high‐NIR photoresponsivity of the heterojunction photodetector is attributable to the efficient electron–hole separation at the gate‐controlled ReS_2_/ReSe_2_ heterojunction. The photocarriers generated by interlayer transition seem to be readily separated by the relatively high internal electric field at the gate‐controlled junction interface.

Subsequently, we analyzed the photoresponsivity of this heterojunction device according to various gate voltage biases (*V*_GS_ = −40, 0, 40 V) and wavelengths of incident lasers (λ = 405, 655, 785, 850, 980, 1310 nm), as shown in Figure 4d. Under the illumination of lasers with short wavelengths less than 850 nm, the ReS_2_/ReSe_2_ heterojunction photodetector exhibited a decreasing trend in photoresponsivity as the gate bias was reduced. Because the effective barrier height at the metal–vdW junction increases as applying a larger negative gate bias, the contact resistance is expected to increase, consequently decreasing the collection probability of photocarriers. On the other hand, when exposed to lasers with long wavelengths greater than 980 nm, the photoresponsivity increased as the gate voltage decreased. This may be because the photocurrent in that wavelength range (greater than 980 nm) was mainly induced by photocarriers generated through the interlayer transition at the ReS_2_/ReSe_2_ heterojunction interface. When a negative gate bias is applied (blue line), as depicted in Figure 4e, the energy band of ReSe_2_ shifts up, increasing the electric field at the junction interface and thereby collecting a greater number of the photocarriers generated at the interface. In contrast, the down‐shifted ReSe_2_ energy band due to a positive gate bias (red line) decreases the electric field at the junction interface so that the collection probability of photocarriers is reduced. Furthermore, we investigated the temporal photoresponse characteristics of the ReS_2_/ReSe_2_ heterojunction photodetector at various wavelengths of incident lasers. Figure 4f shows the temporal photoresponse curves measured under illumination by both visible (λ = 405 nm) and NIR (λ = 1310 nm) light. Here, the measured photocurrents were normalized for analysis of the photoswitching operations. When illuminated under NIR light, a considerably rapid increase and decrease in photocurrents were observed at the rising and decaying edges, respectively. This indicates that the NIR photoswitching operation of the ReS_2_/ReSe_2_ heterojunction device is considerably more rapid than that under visible light. We extracted the rising and decaying times according to the wavelength of the incident light and plotted the data in Figure 4g. The photoresponse times obtained under NIR light illumination (τ_r_/τ_d_: 4/12 ms at 1310 nm) were considerably shorter compared to the other wavelength cases, owing to the more rapid electron–hole separation of the photocarriers generated by the interlayer transition. The photoresponse times under exposure of 405 and 980 nm lights were τ_r_/τ_d_: 2.29/6.95 and 0.103/0.313 s, respectively, because these lights are absorbed by the individual vdW materials through the band‐to‐band transition. We also confirmed that the photoresponse times are affected by the applied gate voltage. As shown in Figure 4h, the rising and decaying times decreased as applying a larger negative gate bias under illumination by both visible (λ = 405 nm) and NIR (λ = 1310 nm) light. This is because the up‐shifted ReSe_2_ energy band due to the larger negative bias increases the electric field at the junction interface, subsequently enabling rapid collection of photocarriers. The minimum rising and decaying times were 1 and 4 ms, which were obtained at *V*_GS_ = −40 V under 1310 nm laser illumination. In Figure S4 of the Supporting Information, we have confirmed the stability of the ReS_2_/ReSe_2_ heterojunction photodetector with respect to the repetitive photoswitching cycle. Additionally, we investigated the changes in the photoresponsivity values (Figure 4i) and the photoresponse times (Figure 4j) according to incident laser power. An exponential increase in photoresponsivity was observed under all wavelength conditions as the incident laser power decreased, owing to the reduced scattering among the photocarriers.^12^ When the laser with the lowest power (5 pW) was used, the photoresponsivity values were considerably high over the entire spectral range (6.78 × 10^6^ A W^−1^ for 405 nm and 1.58 × 10^5^ A W^−1^ for 1310 nm). In addition, the rising and decaying times were shortened by reducing the incident power for both visible (the lowest λ = 405 nm) and NIR (the highest λ = 1310 nm) light, as shown in Figure 4j. This can also be explained by suppressed photocarrier scattering under low power irradiation. The minimum rising and decaying times were 0.29 and 1.5 ms under illumination by NIR (λ = 1310 nm) light with the lowest power of 5 pW, which were considerably shorter than in the case of visible light with λ = 450 nm (τ_r_/τ_d_: 0.278/1.14 s), owing to the rapid electron–hole separation of photocarriers after the interlayer transition. This excellent NIR photoswitching behavior of the ReS_2_/ReSe_2_ heterojunction device is better than that of previous NIR photodetectors fabricated on other vdW materials, such as graphene (τ_r_/τ_d_: >50/>50 s),^4^ ternary metal chalcogenides (τ_r_/τ_d_: 3.0/3.3 s),^23^ and BP (τ_r_/τ_d_: 1/4 ms).^15^ In addition, the other important optoelectronic parameters,^38^, ^39^, ^40^ such as detectivity and external quantum efficiency, were also extracted from the photodetectors fabricated on the ReS_2_, ReSe_2_, and ReS_2_/ReSe_2_ heterojunctions; the plotted data is provided in the Supporting Information chapter (Figure S5, Supporting Information). Finally, for performance comparison of the gate‐controllable ReS_2_/ReSe_2_ heterojunction photodetector with other devices, we plotted the photoresponsivity values obtained in this study and previous studies for vdW photodetectors^4^, ^5^, ^6^, ^7^, ^8^, ^9^, ^10^, ^11^, ^13^, ^23^, ^41^, ^42^, ^43^ in Figure 4k. Our gate‐controllable ReS_2_/ReSe_2_ heterojunction photodetector (blue dotted line) exhibited relatively high photoresponsivity values over a broad range of wavelengths, compared to other vdW photodetectors. In particular, while most of the previous studies exhibited difficulty in achieving IR light detection successfully, our photodetector exhibited an outstanding photoresponse under NIR (λ = 980 and 1310 nm) light illumination. We note that the photoresponsivity values that appear in Figure 4k were obtained under similar laser power conditions of ≈1 nW (Table S1, Supporting Information) to increase the reliability of the comparison.

## 3 Conclusion

We presented a highly efficient NIR photodetection based on the interlayer optical transition phenomenon in a vdW heterojunction structure consisting of ReS_2_ and ReSe_2_. In particular, by applying a new gate terminal to the two‐terminal vdW heterojunction photodetector, we controlled the heterojunction interface and consequently achieved high photoresponsivity over a wide detection range. First, we investigated the role of metal contacts (Ti and Pt) on the device performance of single‐vdW‐material‐based photodetectors (ReS_2_ and ReSe_2_). By using Ti contacts on the vdW materials, we achieved a high photoresponsivity (3.23 × 10^6^ A W^−1^ for ReS_2_ and 1.13 × 10^4^ A W^−1^ for ReSe_2_); however, the photoresponse was slightly slow (rising/decaying times: 8.74/13.1 s for ReS_2_ and 5.20/5.30 s for ReSe_2_). In contrast, the Pt‐contacted devices exhibited a relatively fast photoresponse (rising/decaying times: 4.94/3.20 s for ReS_2_ and 0.032/0.053 s for ReSe_2_) and low photoresponsivity characteristics (1.70 × 10^4^ A W^−1^ for ReS_2_ and 6.12 × 10^2^ A W^−1^ for ReSe_2_), owing to the high contact resistance between Pt and the vdW materials. In addition, the photodetection ranges of the ReS_2_ and ReSe_2_ devices were limited by their energy bandgap properties (up to 850 nm for ReS_2_ and 980 nm for ReSe_2_). By exploiting these single vdW materials, we then implemented a vdW heterojunction with a staggered bandgap alignment that induces an interlayer optical transition; this bandgap alignment was experimentally confirmed by KPFM analysis. The use of this vdW heterojunction structure resulted in the extension of the previous photodetection range up to the NIR region (λ = 1310 nm). Furthermore, by applying an additional gate terminal to the two‐terminal vdW heterojunction photodetector, we could control the heterojunction interface and thereby achieve excellent photoresponsivity over a broad detection range (6.78 × 10^6^ A W^−1^ for 405 nm and 1.58 × 10^5^ A W^−1^ for 1310 nm). This study on a gate‐controllable vdW heterojunction is expected to offer a novel device platform for achieving high‐performance IR photodetectors.

## 4 Experimental Section

*Fabrication of Device*: Mechanically exfoliated ReS_2_ and ReSe_2_ layers were transferred onto the 90 nm thick SiO_2_ on a heavily boron‐doped Si substrate. The source and drain electrode regions (channel length and width are both 5 µm) were patterned using an optical lithography process. Then, 10 nm thick Ti (for low work function metal contact) or Pt (for high work function metal contact) and 40 nm thick Pd were deposited by an e‐beam evaporator.

*Characterization of Electronic Device*: Using a Keysight B2912A precision source/measure unit, the current–voltage characteristics (*I*_D_–*V*_D_ and *I*_D_–*V*_G_) of the devices were measured. From the obtained *I*_D_–*V*_G_ curve, the threshold voltage (*V*_TH_) and on‐current (*I*_on_) values were extracted. Here, all drain currents were normalized by the channel width (*W*_ch_ = 5 µm).

*Characterization of Optoelectronic Device*: To measure the photocurrent (*I*_ph_) of the devices, the channel region of the devices was illuminated by dot‐shaped laser sources with a power density of 4 mW cm^−2^ and wavelengths of 405, 655, 785, 850, 980, and 1310 nm. The photoresponsivity (*R*) values were extracted through the equation *R* = *I*_ph_/*P*_laser_, where *I*_ph_ and *P*_laser_ represent the generated photocurrent and the total power of incident laser, respectively. Moreover, to investigate the temporal photoresponse characteristics, the laser source was lighted up according to a photoswitching cycle (20 s of on‐state and 20 s of off‐state). Then, the rising time (τ_r_) was extracted from 10% to 90% of the measured maximum photocurrent value. The decaying time (τ_d_) was similarly measured vice versa (time taken from 90% to 10%). The extraction method for the rising and decaying times is explained in detail in Supporting Information (Figure S3, Supporting Information).

*Fabrication of vdW Heterojunction Device*: A poly(methyl methacrylate) (PMMA) layer and a polyvinyl alcohol (PVA) layer were coated onto a Si substrate. Then, the ReS_2_ layer was exfoliated onto the PMMA/PVA/Si substrate. Using the mechanical transfer process, the ReS_2_ layer on PMMA/PVA/Si substrate was transferred onto the ReSe_2_ layer on SiO_2_/Si substrate that was prepared in advance. Finally, optical lithography and deposition processes were performed to fabricate the source and drain electrodes (10/40 nm of Ti/Pd).

*Raman and KPFM Analyses of vdW Heterojunction*: After forming the heterojunction structure of ReS_2_ and ReSe_2_, Raman spectroscopy (Alpha300 M+, WITec) was performed at three different points: (i) ReS_2_, (ii) ReSe_2_, and (iii) the overlapped area of ReS_2_/ReSe_2_. For the Raman measurement, a laser with a wavelength of 532 nm, a power of 2 nW, and a beam size of 0.7–0.9 µm was used. The work function mapping images and the line profiles of the ReS_2_/ReSe_2_ heterojunction were measured through KPFM analysis. Prior to KPFM measurement, the Pt/Ir‐coated Si tip was calibrated on a HOPG surface. Using the well‐known work function of the HOPG (*W*_HOPG_ = 4.6 eV), the work function of the KPFM tip (*W*_tip_ = 4.92 eV) was calculated (*W*_tip_ – *W*_HOPG_ = Δ*V*_CPD_HOPG_). Then, the work function of the vdW material (*W*_vdW_) was evaluated (*W*_tip_ – *W*_vdW_ = Δ*V*_CPD_vdW_) by measuring the contact potential difference (Δ*V*_CPD_vdW_) between the KPFM tip and the surface of the vdW material.

## Conflict of Interest

The authors declare no conflict of interest.

## Supporting information

Supplementary
