# Supplementary material for: Highly Efficient Infrared Photodetection in a Gate‐Controllable Van der Waals Heterojunction with Staggered Bandgap Alignment
Source: Adv Sci (Weinh). 2018 Jan 18;5(4):1700423. doi: 10.1002/advs.201700423 (PMC5908375; doi:10.1002/advs.201700423)
Supplement: Supplementary file 1 — Supplementary [file ADVS-5-1700423-s001.pdf]

Copyright WILEY-VCH Verlag GmbH & Co. KGaA, 69469 Weinheim, Germany, 2017.

Supporting Information

for *Adv. Sci.,* DOI: 10.1002/advs.201700423

Highly Efficient Infrared Photodetection in a Gate-Controllable Van der Waals Heterojunction with Staggered Bandgap Alignment

*Seo-Hyeon Jo, Hae Won Lee, Jaewoo Shim, Keun Heo, Minwoo Kim, Young Jae Song, and Jin-Hong Park**

Extraction of the effective barrier height (Φ_{eff}) at the junction between metal and vdW material

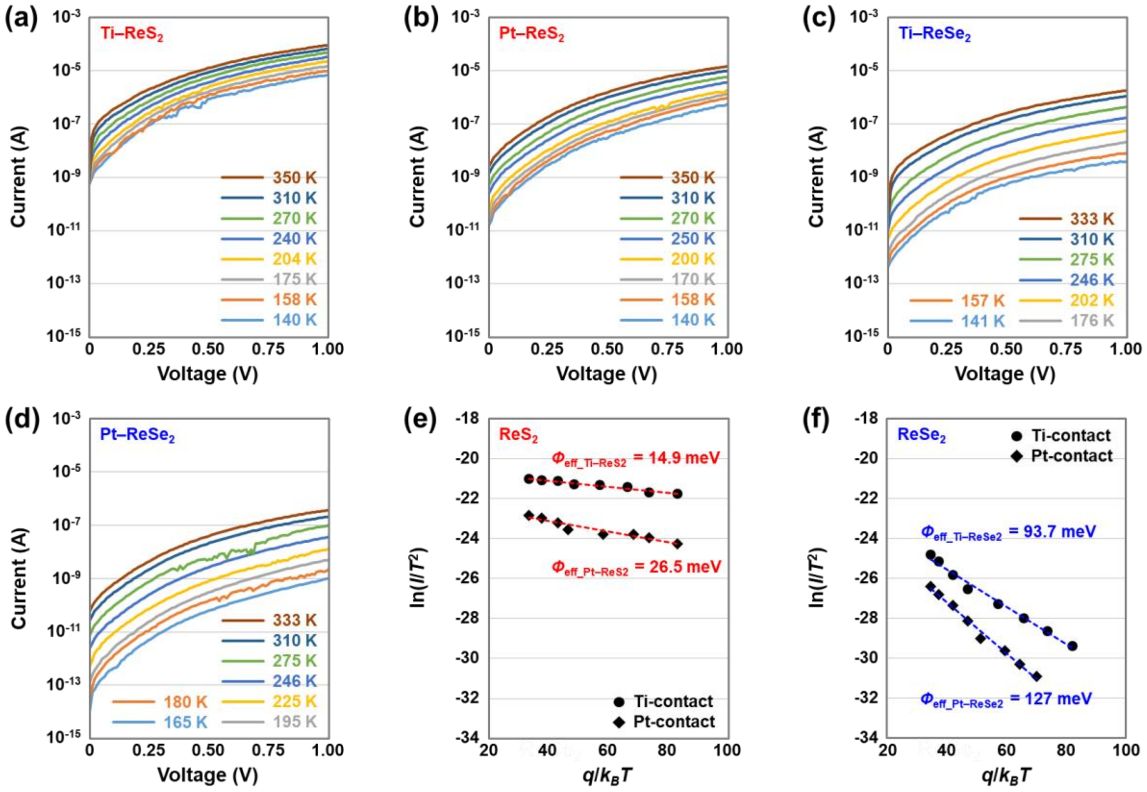

Figure S1. Current–voltage characteristics of (a) Ti–ReS_2, (b) Pt–ReS_2, (c) Ti–$ReSe_2$, and (d) Pt–$ReSe_2$ junctions at various temperatures. Richardson plot ($\ln(I/T^2)$ versus q/k_BT) for (e) ReS_2 and (f) $ReSe_2$-based metal–vdW material–metal (MVM) junction devices at 1 V.

To analyze the difference in barrier heights between metals (Ti and Pt) and vdW materials (ReS_2 and $ReSe_2$), we performed temperature-dependent electrical measurements on the metal–vdW material–metal (MVM) junction devices and extracted the corresponding effective barrier height values. Figures S1a–d show the *I*–*V* characteristics of the MVM junction devices at various temperatures. Then, we estimated the effective barrier height (Φ_{eff}) values at the junction between the metal and vdW material by considering a conventional thermionic emission current model; this equation can be written as

$$I = AA^{*}T^{2}\exp\left(-\frac{q\phi_{eff}}{k_{B}T}\right)\left[\exp\left(\frac{qV}{k_{B}T}\right)-1\right], \quad \text{(Supplementary Equation 1)}$$

where A is the junction area, A^* is the effective Richardson constant, T is the temperature, q is the elementary charge, k_B is the Boltzmann constant, and V is the applied voltage. As shown in Figures S1e and f, the plots of $\ln(I/T^2)$ versus q/k_BT were used in the estimation of the

effective barrier height at metal–vdW junctions. The extracted slopes of the trend lines (dotted line) indicate the corresponding effective barrier heights.

Energy band diagrams for the metal–vdW mateiral–metal (MVM) junction

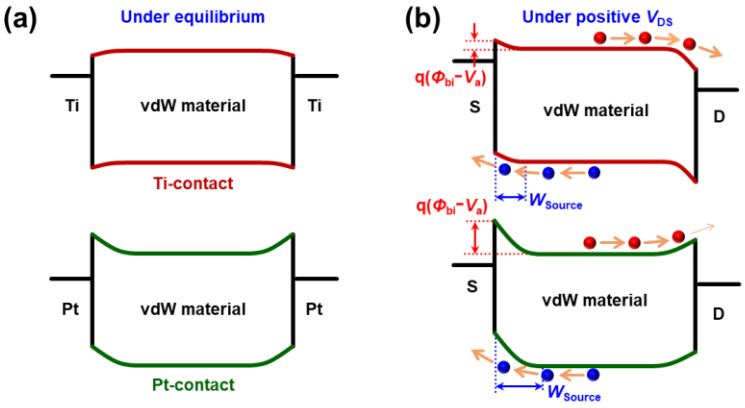

Figure S2. Energy band diagrams for the Ti–vdW–Ti (red line) and Pt–vdW–Pt (green line) junctions under the conditions of (a) an equilibrium state and (b) application of a positive drain voltage (V_{DS}).

As shown in Figure S2, we predicted the energy band alignments of the metal–vdW material–metal (MVM) junctions following initial contact with Ti and Pt electrodes. Under the equilibrium state (Figure S2a), a relatively high electron barrier height is expected in the Pt-contact case because of the higher work function of Pt ($W_{Ti} < W_{Pt}$). After applying a positive V_{DS} (Figure S2b), although the photoexcited holes in the source–vdW junctions are easily collected in both cases (Ti and Pt electrodes), it is relatively difficult to collect the photoexcited electrons in the vdW–Pt junction because of the potential barrier located on the right side. Therefore, as compared to the Pt-contact case, the Ti-contact device is predicted to yield a higher photoresponsivity.

In terms of the collection speed of photocarriers, we should further consider the width of the depletion region at the metal–vdW junctions. This is because the photoexcited carriers are much more rapidly separated by the local electric field in the depletion region than the bulk channel region. As shown in Figure S2b, because the potential barrier at the Pt–vdW junction is larger than that at the Ti–vdW junction, the Pt–vdW junction forms a wider depletion region ($W_{Source} = [2\varepsilon_s(\Phi_{bi} - V_a)/qN_d]^{1/2}$). Therefore, it is relatively faster to collect the photocarriers in the Pt-contact devices.

Photoresponse (rising and decaying) time extraction in the photodetector

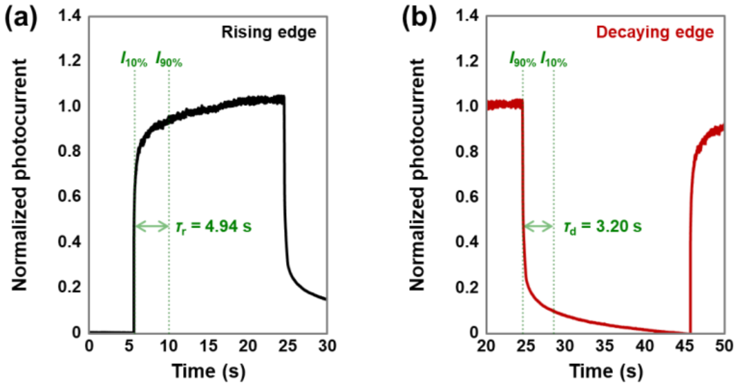

Figure S3. (a) Rising and (b) decaying edges of the temporal photoresponse curve obtained for the Pt-contacted ReS_2 photodetector. Here, the laser switching cycle was 20 s and the measurements were performed at a sampling rate of 1 kHz.

To investigate the temporal photoresponse characteristics, the laser source was lighted up according to the photoswitching cycle (20 s of on-state and 20 s of off-state). Then, the rising and decaying times were extracted between 10% and 90% of the photocurrent on the rising and decaying edges, respectively.[S1] For example, the rising time (τ_r, time from $I_{10\%}$ to $I_{90\%}$) and decaying time (τ_d, time from $I_{90\%}$ to $I_{10\%}$) were obtained as 4.94 and 3.20 s, respectively, for the Pt-contacted ReS_2 photodetector, as shown in Figure S3a and b.

10-cycle temporal photoresponse characteristics of the photodetectors fabricated on ReS_2, $ReSe_2$, and the $ReS_2/ReSe_2$ heterojunction

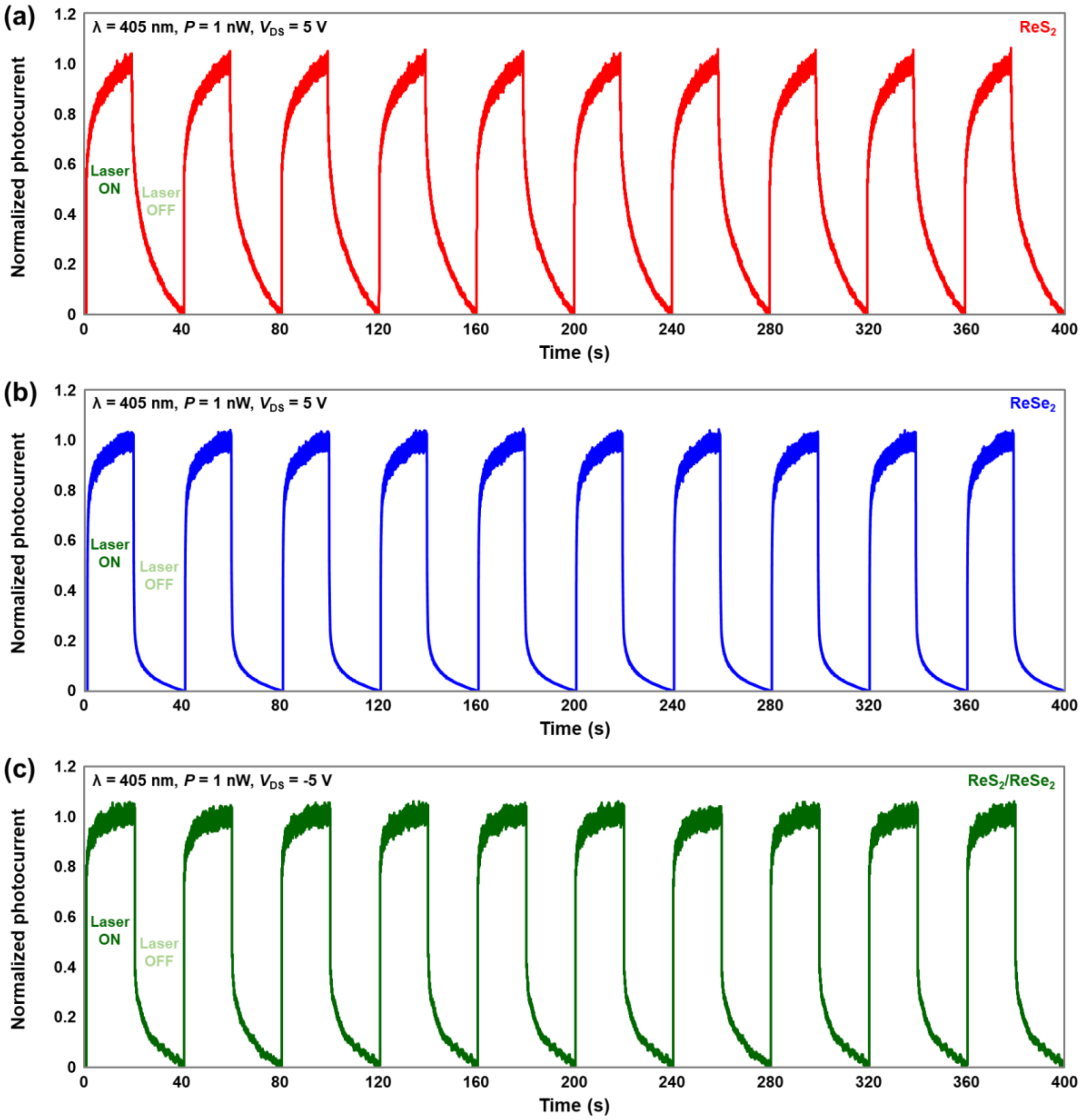

Figure S4. 10-cycle temporal photoresponse curves obtained in the photodetectors fabricated on the (a) ReS_2, (b) $ReSe_2$, and (c) $ReS_2/ReSe_2$ heterojunctions.

To investigate the stability of photodetectors, a 405-nm laser was irradiated for ten photoswitching cycles (20 s on and 20 s off) as the corresponding photocurrents were measured. As shown in Figure S4, during the ten photoswitching cycles, all photodetectors continuously exhibited characteristics of a photoresponse, demonstrating no obvious degradation in photocurrent.

Characteristics of the photodetectors fabricated on ReS_2, $ReSe_2$, and the $ReS_2/ReSe_2$ heterojunction

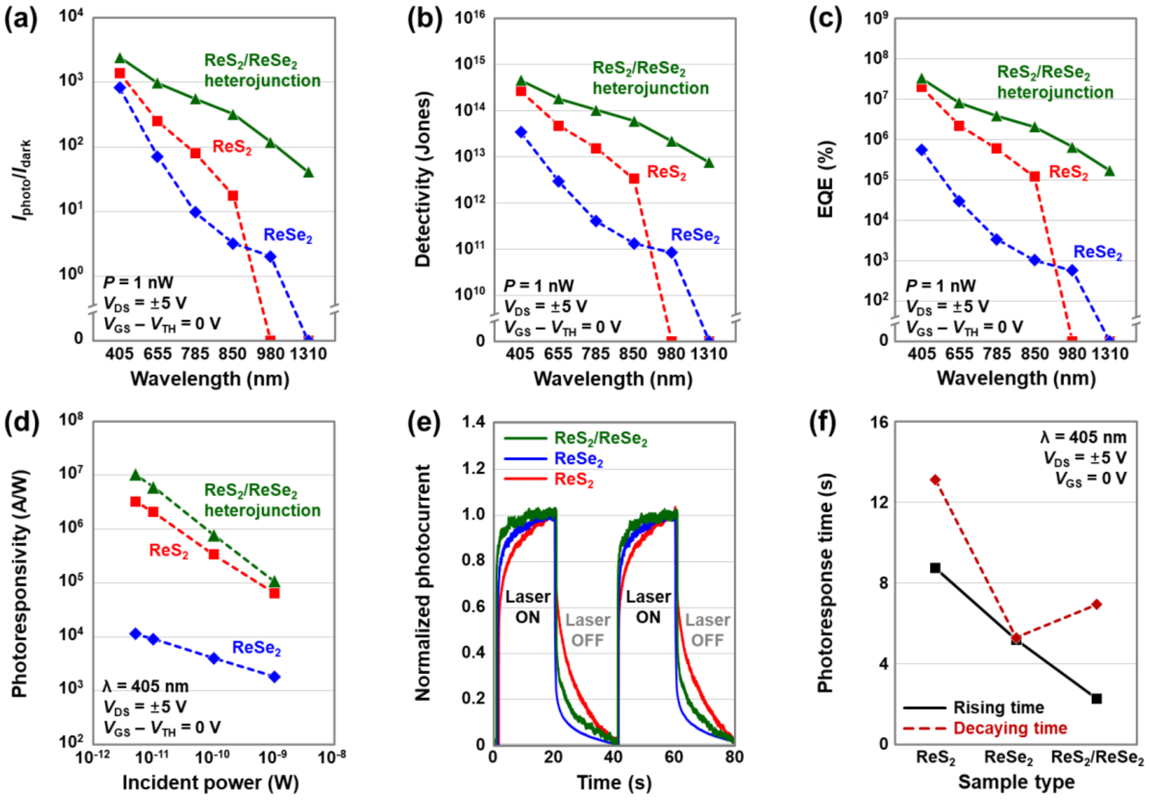

Figure S5. Extracted (a) dark current to photocurrent ratio, (b) detectivity, and (c) external quantum efficiency as a function of the wavelength in the photodetectors fabricated on ReS_2 (red line), $ReSe_2$ (blue line), and $ReS_2/ReSe_2$ (green line) heterojunctions. (d) Photoresponsivity as a function of the incident laser power extracted from the photodetectors fabricated on ReS_2, $ReSe_2$, and the $ReS_2/ReSe_2$ heterojunction. (e) Normalized temporal photoresponse curves and (f) extracted photoresponse times (rising/decaying times) for the ReS_2, $ReSe_2$, and $ReS_2/ReSe_2$ photodetectors.

As shown in Figures S5a–c, we extracted various parameters (dark current to photocurrent ratio, detectivity, and external quantum efficiency) by varying the wavelength of the incident laser. To extract the detectivity (D^*) and external quantum efficiency (EQE), we utilized the following equations:

$$D^* = \frac{R\sqrt{A}}{\sqrt{2qI_{dark}}}, \qquad \text{(Supplementary Equation 2)}$$

$$EQE = R \times \frac{hc}{q\lambda}, \qquad \text{(Supplementary Equation 3)}$$

where R is the photoresponsivity, A is the effective area of photodetector, q is the electron charge, h is the Plank constant, c is the speed of light, and λ is the wavelength of the incident light.[S2-S4] Better performance was confirmed in the $ReS_2/ReSe_2$ heterojunction photodetector as compared to the single-vdW-material-based devices. The highest I_{photo}/I_{dark}, D^*, and EQE values for the $ReS_2/ReSe_2$ heterojunction device were 2.45 × 10^3, 4.53 × 10^{14} Jones, and 3.30 × 10^7 %, respectively, under 405 nm laser illuminations. We also extracted the photoresponsivity values of the $ReS_2/ReSe_2$ heterojunction photodetector as the optical power of the incident laser was varied and compared the obtained values with the photoresponsivity of the devices fabricated on ReS_2 and $ReSe_2$. As shown in Figure S5d, we confirmed a higher photoresponsivity in the $ReS_2/ReSe_2$ heterojunction photodetector, as compared to both the ReS_2 and $ReSe_2$ photodetectors. The maximum value of 1.03 × 10^7 A/W was obtained under 5 pW laser illumination in the $ReS_2/ReSe_2$ heterojunction device. Figures S5e and f present the temporal photoresponse curves and extracted photoresponse times (rising and decaying), respectively, of the ReS_2, $ReSe_2$, and $ReS_2/ReSe_2$ heterojunction photodetectors. Compared to the single-vdW-material-based devices, the photoresponse times were enhanced in the $ReS_2/ReSe_2$ heterojunction photodetector. Exceptionally, the shorter decaying time (τ_{d_ReSe2} = 5.30 s) of the $ReSe_2$ photodetector compared to the $ReS_2/ReSe_2$ device ($\tau_{d\_ReS2/ReSe2}$ = 6.95 s) seems to be caused by the type of chalcogen vacancies in the vdW material, where selenium vacancies act as traps providing a much faster recombination rate for carriers than sulfur vacancies.[S5,S6]

Measurement conditions for vdW photodetectors referred in this paper

Channel material	The number of layers (thickness)	Incident power	Applied bias condition	Reference
**Gate-controllable $ReS_2/ReSe_2$**	**Multi-layer (36.5/50.4 nm)**	**1 nW**	**V_{DS} = –5 V V_{GS} = –22.6 V**	**This work**
Graphene	Mono-layer	1.3 nW	V_{DS} = 20 mV V_{GS} = 0 V	Nat. Commun. 2013, DOI: 10.1038/ncomms2830
Perovskite/MoS_2	Multi-layer (25 nm)	1.57 nW	V_{DS} = 5 V V_{GS} = 20 V	Adv. Mater. 2016, DOI: 10.1002/adma.201600992
O_2-treated ReS_2	Multi-layer (30 nm)	5 nW	V_{DS} = 5 V V_{GS} = 30 V	Adv. Mater. 2016, DOI: 10.1002/adma.201601002
$ReSe_2$	Multi-layer (34.4 nm)	1 nW	V_{DS} = 5 V V_{GS} = –20.8 V	Adv. Mater. 2016, DOI: 10.1002/adma.201601248
MoS_2	Multi-layer (34.3 nm)	1 nW	V_{DS} = 5 V V_{GS} = –36.7 V	Adv. Mater. 2016, DOI: 10.1002/adma.201601248
WSe_2/*h*-BN	Multi-layer (49.6 nm)	1 nW	V_{DS} = 1 V V_{GS} = 0 V	Adv. Mater. 2016, DOI: 10.1002/adma.201600032
Ferro-gated MoS_2	Tri-layer	1 nW	V_{DS} = 5 V V_{GS} = 0 V	Adv. Mater. 2015, DOI: 10.1002/adma.201503340
OTS-doped WSe_2	Multi-layer (27.7 nm)	1.2 nW	V_{DS} = –5 V V_{GS} = 2.4 V	Adv. Funct. Mater. 2015, DOI: 10.1002/adfm.201501170
WS_2	Multi-layer (42 nm)	0.9 nW	V_{DS} = 1 V V_{GS} = 0 V	Sci. Rep. 2014, DOI: 10.1038/srep05209
BP	Multi-layer (8 nm)	2.3 nW	V_{DS} = –1 V V_{GS} = –15 V	Adv. Mater. 2016, DOI: 10.1002/adma.201506352
$Sn(S_xSe_{1-x})_2$	Multi-layer (6 nm)	1.5 nW	V_{DS} = 10 V V_{GS} = 0 V	Adv. Funct. Mater. 2016, DOI: 10.1002/adfm.201600081
GaS	Tri-layer	1 nW	V_{DS} = 2 V V_{GS} = 0 V	Nano. Lett. 2013, DOI: 10.1021/nl400107k
$CuGaSe_2$	Multi-layer (16 nm)	1 nW	V_{DS} = 5 V V_{GS} = 0 V	Phys. Chem. Chem. Phys. 2014, DOI: 10.1039/C4CP02736A
BP/MoS_2	Multi-layer (22/12 nm)	1.5 nW	V_{DS} = 3 V V_{GS} = 0 V	ACS Photonics 2016, DOI: 10.1021/acsphotonics.6b00079

Table S1. Comparison of optical and electrical measurement conditions in studies on vdW photodetectors.

References

[S1] M. Buscema, J. O. Island, D. J. Groenendijk, S. I. Blanter, G. A. Steele, H. S. J. van der Zant, A. Castellanos-Gomez, *Chem. Soc. Rev.* **2015**, *44*, 3691.

[S2] H. Fang, W. Hu, P. Wang, N. Guo, W. Luo, D. Zheng, F. Gong, M. Luo, H. Tian, X. Zhang, C. Luo, X. Wu, P. Chen, L. Liao, A. Pan, X. Chen, W. Lu, *Nano Lett.* **2016**, *16*, 6416.

[S3] H. Tan, C. Fan, L. Ma, X. Zhang, P. Fan, Y. Yang, W. Hu, H. Zhou, X. Zhuang, X. Zhu, A. Pan, *Nano-Micro Lett.* **2016**, *8*, 29.

[S4] L. Ma, W. Hu, Q. Zhang, P. Ren, X. Zhuang, H. Zhou, J. Xu, H. Li, Z. Shan, X. Wang, L. Liao, H. Q. Xu, A. Pan, *Nano Lett.* **2014**, *14*, 694.

[S5] S.-H. Jo, H.-Y. Park, D.-H. Kang, J. Shim, J. Jeon, S. Choi, M. Kim, Y. Park, J. Lee, Y. J. Song, S. Lee, J.-H. Park, *Adv. Mater.* **2016**, *28*, 6711.

[S6] V. Klee, E. Preciado, D. Barroso, A. E. Nguyen, C. Lee, K. J. Erickson, M. Triplett, B. Davis, I.-H. Lu, S. Bobek, J. McKinley, J. P. Martinez, J. Mann, A. A. Talin, L. Bartels, F. Leonard, *Nano Lett.* **2015**, *15*, 2612.
